# Mobile 5P-Medicine Approach for Cardiovascular Patients

**DOI:** 10.3390/s21216986

**Published:** 2021-10-21

**Authors:** Ivan Miguel Pires, Hanna Vitaliyivna Denysyuk, María Vanessa Villasana, Juliana Sá, Petre Lameski, Ivan Chorbev, Eftim Zdravevski, Vladimir Trajkovik, José Francisco Morgado, Nuno M. Garcia

**Affiliations:** 1 Instituto de Telecomunicações, Universidade da Beira Interior, 6200-001 Covilhã, Portugal; hanna.denysyuk@ubi.pt (H.V.D.); ngarcia@di.ubi.pt (N.M.G.); 2 Escola de Ciências e Tecnologias, University of Trás-os-Montes e Alto Douro, Quinta de Prados, 5001-801 Vila Real, Portugal; 3 Centro Hospitalar do Baixo Vouga, 3810-164 Aveiro, Portugal; 72152@chbv.min-saude.pt; 4 Faculty of Health Sciences, Universidade da Beira Interior, 6200-506 Covilhã, Portugal; julianasa@fcsaude.ubi.pt; 5 Centro Hospitalar e Universitário do Porto, 4099-001 Oporto, Portugal; 6 Faculty of Computer Science and Engineering, SS. Cyril and Methodius University, 1000 Skopje, North Macedonia; petre.lameski@finki.ukim.mk (P.L.); ivan.chorbev@finki.ukim.mk (I.C.); eftim.zdravevski@finki.ukim.mk (E.Z.); trvlado@finki.ukim.mk (V.T.); 7 Computer Science Department, Polytechnic Institute of Viseu, 3504-510 Viseu, Portugal; fmorgado@estgv.ipv.pt

**Keywords:** 5P-Medicine, digital health, mobile bio-sensing for medicine, patient empowerment technologies, artificial intelligence, cardiovascular diseases

## Abstract

Medicine is heading towards personalized care based on individual situations and conditions. With smartphones and increasingly miniaturized wearable devices, the sensors available on these devices can perform long-term continuous monitoring of several user health-related parameters, making them a powerful tool for a new medicine approach for these patients. Our proposed system, described in this article, aims to develop innovative solutions based on artificial intelligence techniques to empower patients with cardiovascular disease. These solutions will realize a novel 5P (Predictive, Preventive, Participatory, Personalized, and Precision) medicine approach by providing patients with personalized plans for treatment and increasing their ability for self-monitoring. Such capabilities will be derived by learning algorithms from physiological data and behavioral information, collected using wearables and smart devices worn by patients with health conditions. Further, developing an innovative system of smart algorithms will also focus on providing monitoring techniques, predicting extreme events, generating alarms with varying health parameters, and offering opportunities to maintain active engagement of patients in the healthcare process by promoting the adoption of healthy behaviors and well-being outcomes. The multiple features of this future system will increase the quality of life for cardiovascular diseases patients and provide seamless contact with a healthcare professional.

## 1. Introduction

The health care systems traditionally followed the paternalistic approach [1]. In recent years, there has been a noticeable paradigm shift towards patient- and community-centered strategies, empowering those approaches with modern technologies [2,3,4]. Today, it is impossible to imagine living without ubiquitous technologies such as smartphones and smart devices. All of us are connected using the Internet, and each of our moves is either being recorded or analyzed. Smartphones, sensors, and smart devices also allow measurement of various parameters, contributing to building a general model of our personal and our health care profile [5,6,7]. These and other aspects allow employment of the novel technologies for patient empowerment, in a sense that the patients themselves are directly included in their healthcare management. Healthcare is not an institutional or hospital-based service, but the patients themselves and their communities are at the service center [8]. However, the application of modern paradigms is yet to be studied and employed to their full potential [9]. It is especially true for Cardiovascular patients.

Based on World Health Organization (WHO) data published in 2016, cardiovascular diseases are highly prevalent around the world [10]. Still, the middle- and low-income countries report higher prevalence with significant worldwide rates of morbidity and mortality [11]. Cardiovascular diseases are considered chronic non-infectious diseases related to different risk factors, including arterial hypertension, diabetes, hyperlipidemia, obesity, smoking, an imbalanced diet, and lack of physical activity [12]. The control and treating of these diseases include the normalization of blood pressure, the reduction of stress, healthy nutrition, and the increasing level of physical activity [13].

In general, the technology can be combined for the creation of complex solutions with complex measurements [14]. At this point, the 5P-Medicine concept is essential for the design of solutions to Predictive, Preventive, Participatory, Personalized, and Precision Medicine [15]. Furthermore, it allows the promotion of several actions, prior, during, and after some healthcare problems [16]. Still, the most important is the technology enables medical people to act just in time. Combining the different concepts also allows predicting the future and increasing the effectiveness of treatment to a minimum of 40%.

Nowadays, technology is part of the different daily activities. The various devices include the possibility of connection to the Internet [17]. Most of them also have a set of embedded sensors, including an accelerometer, magnetometer, gyroscope, and microphone, which allows the acquisition of different types of data [18]. In addition, these devices can add connections to other sensors, including electrocardiography, electromyography, pressure sensors, sphygmomanometer, among others, that allow the collection of medical data. It will enable the constant monitoring and the creation of patterns of the different diseases for the acquired data, allowing a remote evaluation of the patients [19]. Technology also allows the communication that promotes telemedicine and telemonitoring, making the patient independent, promoting patient empowerment, and centering the medical treatments in the patient [20].

The main goal of this paper consists in the proposal of a novel system architecture that considers the 5P-Medicine paradigm (Predictive, Preventive, Participatory, Personalized, and Precision Medicine) approach to empowering cardiovascular patients. Currently, the technology is improving, and the different measurements can be performed anywhere with the high commodity for the patients. The secondary endpoints consist of the different prospective achievements needed for the creation of the proposed system:

- Before the planning of the system, analysis of the state-of-the-art about the current applications which use mobile and wearable personal devices for promoting personalized digital health care must be performed.
- Identify the implementation challenges when applying the approach to real implementations.
- Analyze the required device features, because, for the use and implementation of the system, a minimum hardware and software requirements are needed.
- Analyze the required sensors because different sensors are needed for the data acquisition that will help the healthcare professionals in the monitoring of the cardiovascular patients.
- Increase the patients’ autonomy with the easy and seamless contact with healthcare providers and professionals.

This paragraph ends the introduction. In continuation, Section 2 offers state-of-the-art patient empowerment, the 5P-Medicine concept, wearable devices, smart mobile devices, cardiovascular diseases and technology, and bio-signals acquisition and processing. Next, the research background, research design, and expected results are presented in Section 3. Finally, the analysis and further implementation of the system are discussed in Section 4, presenting the conclusions of this paper in Section 5.

## 2. State-of-the-Art

This section started with state-of-the-art representing the current stage of the 5P medicine approach. Section 2.1 aims to identify novel contributions of the 5P medicine to the digital health management practice, in particular for cardiovascular disease. Section 2.2 represented the advanced methods and solutions for empowering patients with different chronic conditions. Section 2.3 captured current trends in the use of mobile and wearable personal devices for monitoring and collecting physiological parameters. Section 2.4 presents the technology to prevent and monitor cardiovascular diseases. Finally, Section 2.5 discussed the most suitable Bio-signals acquisition and processing techniques available in the literature.

### 2.1. 5P-Medicine Concept

More than ten years ago, Leroy Hood defined the concept of 4-P Medicine, i.e., Predictive, Preventive, Personalized, and Participatory [21], to highlight the future change of medical intervention from care to prevention. This concept evolved, with Pravettoni and Gorrini, into a 5-P model that included Psychocognitive medicine [22]. It recognizes that patients have behaviors, habits, and beliefs that influence their interaction with health. Adding this dimension to the biological entity is essential to empower the person to share decisions over his health [22]. The concept of 5-P Medicine includes Predictive, Preventive, Participatory, Personalized, and Precision Medicine [15]. New technologies have significantly developed with eHealth providing solutions to improve healthcare [23]. More recently, the use of mHealth to improve autonomy in the control of chronic diseases. As public health systems are being modernized worldwide, conventional medicine is undergoing a profound transformation, and new digital 5P-based medical models are emerging. It is becoming crucial to identify new disease monitoring and prevention methods using modern information and communication technologies. However, some challenges remain to enhance accessibility, determine the exact impact on health, know the financial consequences, and improve data security [24].

As public health systems are being modernized worldwide, conventional medicine is undergoing a profound transformation, and new digital 5P-based medical models are emerging. Therefore, developing new disease monitoring and prevention methods is crucial using modern information and communication technologies. The goal is to understand and implement how conventional medical approaches and medicine of the future will co-exist and interact.

### 2.2. Patient Empowerment

Health care systems have been shifting their delivery of care towards patient- and community-centered approaches. Shared decisions have increasingly replaced the past paternalistic, and hospital-based healthcare service model, self-care, self-management, and home-based care [25]. WHO advocates patient empowerment as an essential tool to promote health. It is defined as a process to educate and give tools to the patients by healthcare professionals to recognize community and cultural differences and the participation of the patients [26]. The effect of patient empowerment on health results has been studied in several chronic conditions such as diabetes and heart diseases [27,28]. Empowered patients tend to have a better quality of life and well-being [29], impacting health outcomes to be consistently proved.

The use of technology to promote patient empowerment is being widely discussed and analyzed. Systems and tools have been developed to encourage and maintain healthy behaviors, education, and disease self-management. Technological development is expected to reduce financial costs and contribute to the sustainability of health care through rethinking interactions between patients and professionals, overcoming geographical barriers, and developing home-based solutions [30,31]. Several projects developed tools and systems to promote patient autonomy using online platforms and mobile devices in many medical fields. Digital tools to encourage autonomy and treatment guidance not only for chronic diseases, such as respiratory diseases, diabetes, palliative care, and acute infections [32,33,34,35]. The transformation of healthcare and medicine through technology has been predicted for several years. However, current changes in healthcare delivery globally, boosted by the COVID-19 pandemic, promote a vital momentum to drive digital transformation in healthcare [36].

### 2.3. Wearable and Smart Mobile Devices

Researchers and technology companies have explored the use of wearable sensors to monitor physiological parameters and activities for the past decade. These devices can record real-time information through usable gadgets or being incorporated into clothing. They can measure physiological signals, such as heart rate, body temperature, arterial oxygen saturation, breathing rate, and body movement. They also have wireless communication modules integrated with mobile devices [37]. Real-time feedback is useful both for patients and for healthcare professionals. Patients can better understand their disease and see immediate and objective results from their actions, allowing them to improve behaviors and be empowered to make decisions [38]. Health professionals can access individual data to provide personalized advice, predict events, prevent disease, early diagnosis, and chronic control conditions [39]. Of course, these outcomes can only be achieved if data is secure and reliable. However, there are still challenges facing the way sensors and systems are developed.

Additionally, there is also potential to increase sensors and wearables in clinical trials, accelerating knowledge and new treatments. For this, the concern about data safety is essential to improve the acceptance of the systems [40]. Different devices in the market embed or connect to reliable sensors to monitor various health parameters related to cardiovascular diseases [41,42].

### 2.4. Cardiovascular Diseases and Technology

Cardiovascular diseases are highly prevalent across the globe representing 31% of global deaths [10], with half of deaths occurring in the middle- and low-income countries. These diseases are related to unhealthy behaviors and poor control of chronic conditions such as hypertension, diabetes, obesity, and cardiac failure [12].

The cornerstone of cardiovascular diseases management and prevention is based on interventions to motivate lifestyle modification and adherence to effective cardiovascular medications. Successful strategies to promote smoking cessation, increase physical activity levels, encourage a healthy diet, and improve medication adherence are associated with improvements in morbidity and reductions in mortality [43,44]. However, given the millions of people at risk for or with cardiovascular diseases, there are practical, logistical, geographical, and financial challenges in delivering comprehensive risk factor management to diverse populations. Health systems worldwide are charged with finding ways to reach more people in efficient and scalable ways.

The use of technology to prevent and monitor cardiovascular diseases has been tested with positive results [45,46], leading to new clinical practice recommendations [47]. Moreover, there is evidence of the need to develop these tools to achieve more accurate results and disseminate their adoption [48].

### 2.5. Bio-Signals Acquisition and Processing

The data acquisition and processing of sensors’ data have been studied in the literature [49,50,51]. They consist of the instrumentation of the different individuals with wearable and smart mobile devices connected to other external sensors to increase the data acquisition capabilities [52,53,54]. Various studies use cloud servers to store the data acquired in natural environments [55,56,57,58]. In general, acquiring the data is also part of a system that includes data processing, cleaning, imputation, fusion, and classification [59]. The data cleaning mainly consists of removing the noisy data for the correct perception of the data acquired with different methods, including low-pass filter and high-pass filter [52]. The data imputation measures the data that failed in the data acquisition process. Different methods can be implemented, including K-Nearest Neighbors imputation [60]. The feature extraction consists of analyzing the data related to cardiovascular diseases, including the heart rate, heart rate variability, different variables, and measurements associated with the QRS complex, and other measures [61]. However, one sensor/variable is not sufficient for complex measurements, and the fusion of the different data must be performed [62,63,64]. The final stage consists of the data classification. It may include various AI techniques, including Artificial Neural Networks, Support Vector Machines, Decision Trees, and Ensemble Learning algorithms [65]. Finally, the different measurements and machine learning methods will be more accurate using the Big Data concept in healthcare [66,67,68,69].

## 3. Methods and Expected Results

This section is the main section of this scientific paper, presenting the research background in Section 3.1. Next, Section 3.2 presents the different stages of the design of the proposed system. Section 3.3 presents the different methods that will be expected to be used for the measurement of patients’ empowerment. Finally, Section 3.4 presents an overview of the expected results to be obtained with the proposed system.

### 3.1. Research Background

**Figure 1:**
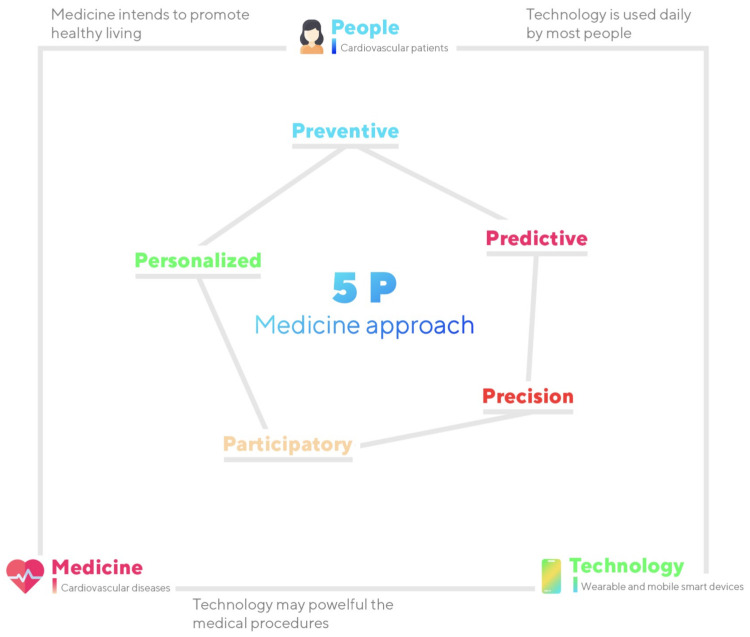
Workflow of the proposed system.

The proposed approach, presented in Figure 1 intends to give a solution that implements the 5P-Medicine paradigm [15], including Precision, Predictive, Preventive, Participatory, and Personalized Medicine related to cardiovascular diseases. For the final implementation, each of the 5Ps implementations will be researched for the final combination. This study aims to integrate the knowledge of different sciences, including computer, mathematics, and medical sciences, to research, develop, validate, and disseminate the developed technological solution.

For this purpose, there are different solutions available in the market. Still, no one includes the different concepts proposed in this paper. We intend to test the proposed system with cardiovascular patients, where the data acquisition will be performed with various sensors available in the market, including sensors available in mobile devices, sensors that can be connected to a BioPlux device [70], and others. These sensors have easy positioning, and all people may use these sensors to acquire health parameters. Still, some of these sensors are expensive. Finally, it includes the connection to a server for further analysis by medical people. In addition, the system must allow communications between healthcare professionals and patients.

The design and development of the proposed solution will consist of executing the main tasks, such as planning and development of data acquisition methods with the technological devices and collaboration of professionals, execution of data acquisition process for the creation of a database with different kinds of data, data analysis for the design of models for each of the 5P-Medicine, and integration of all developments.

The proposed system will be divided into five main parts consisting of each of the different concepts, including Precision, Predictive, Preventive, Participatory, and Personalized medicine. Then, as expected results, a system that integrates all the concepts will be presented in a solution that implements the 5P-Medicine paradigm for cardiovascular diseases.

Following the first P related to Prediction Medicine, the data acquired from the various sensors and wearable and smart mobile devices will be treated to create a method to predict future events or healthcare problems. It consists in utilizing the cloud computing tools for the expected model development. After being prepared, data set integration, the machine learning algorithms, modeling technique, and test design will be applied. Finally, the performance of predictive training models for cardiovascular diseases will be assessed to ensure the quality and reliability of the results. The metrics are numerical measures obtained from the confusion matrix that quantify the performance of a given classifier.

Following the second P related to Prevention Medicine, the medical and clinical health-related data sets, which are the prime concerns in healthcare decision-making, will be used on preventive models development. In addition, these models will be augmented with additional non-clinical background features to enhance the predictive capabilities of the preventive models [71]. With the method developed, the goal is to change people’s behaviors or take adequate actions before healthcare problems occur.

Following the third P related to Participatory Medicine, the patient is the center of the system that will be part of the system. The patient must report important information in the system. The patient will be involved in the system’s further development, where the decision is centered on the patient involved in the treatment and accompanying processes.

Following the fourth P related to Personalized Medicine, the system uses the sensors’ data and the patients to adapt the intervention, recommendations, medication changes, and adequate medical exams. The monitoring and evaluation of the patient’s process must be continuous, with no particular time for the decision-making. It is helpful because it gives independence to the patient. The system performs different measurements when the patients are in their regular environments, adapting their daily living approach. The patients’ examination in their environment will be more reliable because they are examined in their daily living activities. Thus, the system will be integrated into the patients’ living.

Finally, the fifth P, related to Precision Medicine, is intended to create an algorithm capable of predicting the moment and the healthcare problems with high accuracy and exactness.

### 3.2. Research Design

**Figure 2:**
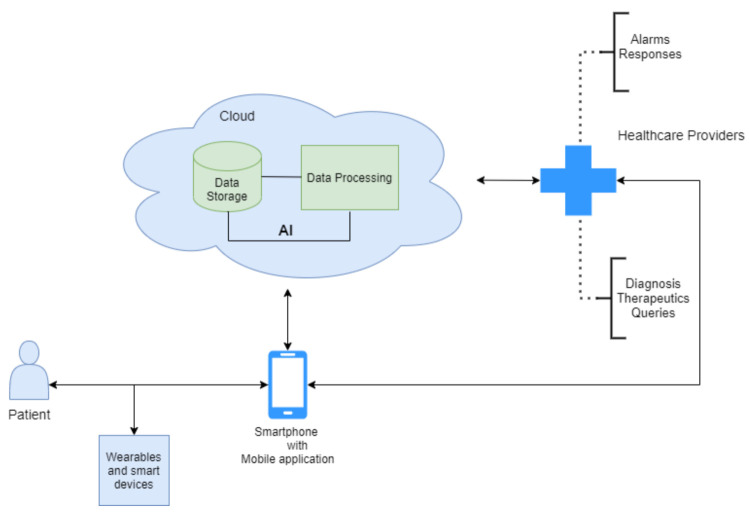
Architecture of the proposed system.

Based on the research background presented in Section 3.1, a global system was designed for the analysis of the data collected by the different sensors available in mobile and wearable devices, which will be sent to the cloud for further processing and generation of the notification to the patients and health systems. The whole design of the proposed system is presented in Figure 2.

**Figure 3:**
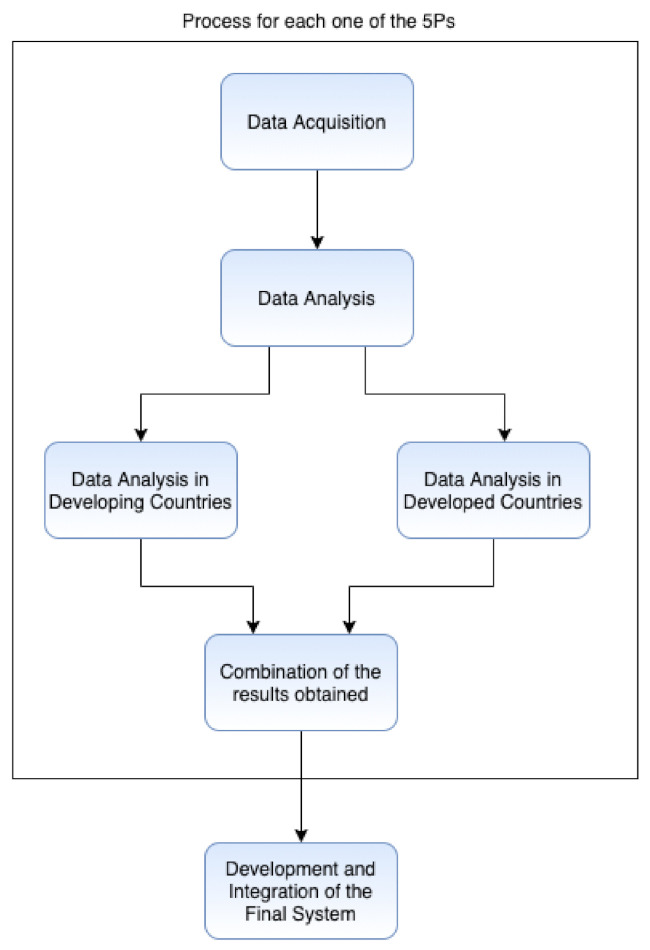
Iteration for the development of the proposed system.

This study will be performed in five different iterations composed of different stages, presented in Figure 3 for each one of the 5Ps. In the following subsections, each of the parts for the research and development of the system will be presented. The proposed system will follow the Regulation (EU) 2017/745 of the European Parliament and the Council established for medical devices.

#### 3.2.1. Data Acquisition

Smartphones and connected wearable devices can quickly generate and collect a large-scale amount of diverse, complex, and dynamic data for analysis. In addition, these technologies are more economical and time-saving than traditional research mode since the entire process can be done remotely, anytime, and everywhere.

Gathering and analysis of clinical information will be performed with support from medical experts. This process involves human participants with cardiovascular diseases. Participation in the study is voluntary, and there is no compromise of the clinical diagnosis or management if the patient does not want to participate. Processing personal data will be carried out in compliance with the General Data Protection Regulation (GDPR), the data protection law of the European Union.

It consists of collecting medical data, including Electrocardiography (ECG) data, bioimpedance data, location data, personal data, data related to the various diseases of the participants, and data acquired from different sensors. The used sensors may be inertial, acoustic, and imaging, stored in a secure server.

The conditions to participate in the study are related to cardiovascular diseases, distributed equally by gender, and different age groups. A waiver of informed consent will be requested for patients in the hospital without conditions to consent and, when possible, given to the patient to sign afterward, as this is a non-experimental study. In the event of death or not signing the informed consent, the legal representative or the closest family members will be asked to determine the patients’ participation in research studies, as it is a study of non-experimental character. In addition, the participants must have an Internet connection available to store the collected data remotely. The acquired data include vulnerable individuals, including older adults, limited capabilities, and other vulnerable people. The study consists of intervention activities by medical doctors for the acquisition of imaging data (or other types of data with medical equipment) to be sent to the data processing stage to control the treatments’ evolution. As it involves different countries, the data from EU and non-EU countries will be imported/exported for further processing by the various technological and medical teams.

Before acquiring the different datasets related to each of the 5P-Medicine concepts, the analysis of the existing methods is performed for the correct planning of the data acquisition process. As the system must be patient-centered, the planning stage is crucial for appropriately developing the technique for data acquisition. Finally, the patients with cardiovascular diseases and group control of healthy people will be recruited.

The positioning of the sensors and the positioning of the mobile device are widely essential for the correct data acquisition, and the different constraints, including environmental and sensor conditions, affect the data acquisition process. Therefore, it can be considered as a limitation of this stage. Another limitation is the definition of the timeline for the acquisition using the device because these devices have limited memory, power processing, and battery capabilities. The research is intended to use open-source development. Thus, the data acquisition will be developed for mobile devices with the Android operating system for smartphones and smartwatches. The smartphones will collect the data from the other devices over-the-air.

The study protocol was approved by the Ethics committee of Universidade da Beira Interior, Covilhã, Portugal, with the reference CE-UBI-Pj-2021-041:ID969.

#### 3.2.2. Data Analysis

It consists of analyzing the data acquired based on the information provided by medical people. It includes artificial neural networks, statistical, and other computational methods to analyze the data. The analysis of the data occurs after the data acquisition.

Before starting the study, the participants fill on a questionnaire related to personal data, i.e., age, gender, diseases, habits, location, biometric data, health data, and other personal data that may be used for the analysis comparison of the different participants on the study. However, the data are always anonymized for technical analysis. The participant is only identified by one ID attributed by the healthcare professional in the study’s invitation, which is only known by the healthcare professional. This data are only used to support the study results, and it will never be used to identify the participants for the scientific community.

Before analyzing the different datasets related to each 5P-Medicine concept, the definition of the possible measurements is needed with the support of medical people. The correct measurements have been essential as the beginning for the excellent performance of the data analysis. It is predicted that a possible limitation is that the data collected is not known, and several experiments are needed before the definition of the concrete variables to analyze. The analyses will be performed with open-source programming technologies, and only some statistical analysis will require more robust software.

#### 3.2.3. Data Analysis in Developing Countries

It consists of analyzing the data acquired in developing countries based on the information provided by healthcare professionals, patients’ lifestyles, and healthcare and environmental conditions for data acquisition of the people from these countries. The developing countries are particular, and they need to be analyzed separately because the people’s characteristics are different from developed countries.

#### 3.2.4. Data Analysis in Developed Countries

It consists of analyzing the data acquired in developing countries based on the information provided by healthcare professionals, patients’ lifestyles, and healthcare and environmental conditions for data acquisition of the people from these countries. The developing countries are particular, and they need to be analyzed separately because the people’s characteristics are different from developing countries.

#### 3.2.5. Development and Integration of Final System

This stage involves developing and integrating several self-contained components such as wearable personal devices connecting for data acquisition, biometrics signal processing, and machine learning algorithms utilizing the cloud computing tools for data processing and model development. As a result, the system will advance the smart solution in personalized and precision eHealth. Furthermore, its innovative solutions will increase the quality of life for cardiovascular diseases patients and be carried out by healthcare professionals, contributing to 5P-Medicine.

### 3.3. Methods for Patient Empowerment and System Analysis

Regarding measures improvements for patient empowerment and system effectiveness, the quantitative and qualitative methods will be applied. In the literature, many authors defended that patients’ active engagement through digital tools in their health could increase self-monitoring effectiveness and empowerment, improving and maintaining a healthier lifestyle.

As engagement is important for long-term use, it is crucial to design technology in such a way that the chance of engagement is high [72]. One way to improve engagement is by incorporating a combination of persuasive design and behavioral change techniques [73]. The combined term for this is persuasive features. Another factor that influences engagement is usability. Usability relates to the quality of the technology in terms of easiness to learn and use it [74]. Usability and engagement together form the user experience. Usability and persuasive features will have a small direct influence on the adherence but will mainly affect engagement. High engagement is likely to improve adherence.

The log data analysis is a quantitative study. Log data analysis can provide information about the use of the technology for numerous users without interfering with normal behavior [75]. Although log data analysis can show interesting differences, it often remains unclear why these differences in use exist. Qualitative studies can only reach some total users, but it gives more insight into barriers and facilitators [76]. The qualitative method is the usability tests and interviews. In this study, the log data analysis will be provided insight into general use and difference in use from the proposed system for a specific time. The usability tests and interviews will be held after the log data analysis.

The availability of more information about the facilitators will motivate patients into adherent behavior. It is important to achieve effective long-term usage of our proposed system for cardiovascular patients. Giving the patient more suitable and credible information, instant support, and feedback by a health care provider about their health will ensure an opportunity to increase their motivation for self-management capability.

### 3.4. Expected Results

After all the developments and tests, it is expected to be available a system for implementing the different concepts of 5P-Medicine for the accompanying, monitoring, and empowerment of cardiovascular patients. Furthermore, the system is expected to be accepted by the medical communities integrated with this system’s design. The system will have the opportunity to be integrated into the National Health System to expand its use and empowering patients and healthcare professionals to achieve better health outcomes.

## 4. Discussion

Implementing a solution that empowers cardiovascular patients based on the 5P-Medicine concept will promote combining technology and medicine. It is an innovative system, and the exploration of the digitalization of the 5P-Medicine is only in the beginning, and further developments are needed. However, several challenges are hard to solve due to the different devices’ characteristics and variety for medical purposes. One main reason for these challenges is the complexity of these developments, and the challenges of long-term and continuous monitoring, sending data and receiving results in real-time.

### 4.1. Multidisciplinary Approach Required

The proposed approach to implementing the treatment and monitoring process between healthcare professionals and computer science experts requires constant communication to plan and develop the final solution. The development of solutions is part of technological people, but knowing the correct collection, data treatment, and purposes is part of the medical people. Therefore, different joint expertise from other people will increase the reliability and validity of the system and future usage. Furthermore, this approach combines various sciences, including medicine, mathematical, and computer science knowledge, making the presented problem fascinating.

### 4.2. Experimental Challenges

The experimental challenges include several variants of potential problems related to the different activities to perform. The limited power processing and battery capabilities are significant challenges that must be considered while developing the software for experimental procedures using lightweight technologies. The next challenge consists of correctly positioning the sensors for accurate data acquisition, where the correct information must be provided to the patients by the mobile application. Furthermore, the connection between different devices and sensors must be available for proper data acquisition. The various connections must be inspected before the experimental data acquisition to avoid this problem. Another challenge consists in the Internet connection needed to store the acquired data into the cloud server. In this case, the system for obtaining the data must store the received data in offline mode to the mobile device’s memory, and it will be sent to the cloud server when an Internet connection is available. The correct positioning of the sensors also reduces the data noise. During the data acquisition, the sensors may have failures that must have reduced effects with the proper modeling of the system.

### 4.3. Complexity and Specificity of System

Following the challenges with the sensors’ data acquisition presented as experimental challenges, other challenges are related to the other components of the proposed system. Thus, several specificities of the system will increase the complexity of the system:

- *Data acquisition and processing:* It must have low latency times to acquire and process data. The frequencies of data acquisition from the different sensors/equipment must be adjusted to obtain better results.
- *Data processing:* The time for data processing can sometimes be high, and the solution’s development time is affected. The nonexistence of rules for analyzing the data is another problem related to data processing, where the data should be tested with different processing methodologies.
- *Data fusion:* The acquired data have different natures, and the complexity of the data fusion and processing may be adapted to the different kinds of data. Various sensors retrieve distinct types of data.
- *Amount of data:* The proposed approach must be prepared to transmit and store a large amount of data related to different sensors.
- *Interaction between sensors and patients:* The patients must be taught and familiarized with the different sensors and mobile devices before using the system.
- *Acquisition of health parameters:* As the proposed approach is related to the acquisition of health parameters, the system must consider different rules for data protection and security during the data transmission and analysis.
- *Patients:* The proposed approach must be adapted to the patients. The data acquired from the different patients must be anonymized and labeled for the healthcare professional’s knowledge responsible for the patient. Only features related to the different data types must be processed because the different data types may identify the patients. Finally, the time zones of the various countries must be controlled to synchronize the results obtained and contacts with different intervenients.
- *Features extracted from the data acquired:* During the development and testing of the developed methods, the best set of features must be identified to increase the results’ accuracy.
- *User interface:* The user interface of the proposed approach must be user-friendly for the different ages of the people as the movements for older adults are more limited than children.

### 4.4. Modeling Challenges

The proposed approach includes technological and medical people from different countries and patients from the selected countries. The main challenge with this system is the existence of varying healthcare diseases related to cardiovascular problems that healthcare professionals must have previously identified. Furthermore, the data acquisition and processing methods must be planned with the knowledge about the diseases [77]. Furthermore, the algorithms for the automatic analysis must be developed for the different disorders. Moreover, the developed methods must be lightweight as preferable or executed in a remote server with the data sent by an Internet connection.

This approach consists of implementing a system that aggregated the measurements related to each of the 5P-Medicine concepts [15]. The method may have different specificities for each development stage that the literature must discover, and the nature of the sensors used healthcare professionals’ knowledge.

The most important piece of the proposed system is the patient, and the developed system must be intelligent to be adapted to the different patients. Therefore, the system’s adaption is crucial for the patient, and it is also vital for the healthcare professionals with a suitable communication method between them. The system must be comfortable for the patient to increase its use. As it includes communication, all the information must be securely stored and transmitted, and the different procedures on the system must be performed in an authenticated mode. Finally, the system should notify the patient of different things in a non-intrusive method. The patients will have the opportunity to have contact with healthcare professionals from other countries. Still, the system must take into account the different time zones to schedule the meeting.

### 4.5. Integration with Real World

After the development and testing of the system, another challenge is integrating the system with the National Health Systems. Changes must be made in the National Health Systems, or adapters should be implemented to allow a seamless operation of both systems and achieve a wide usage of the proposed approach.

However, as this system is developed with different countries’ cooperation, multilingual support must also be implemented in each nation. Furthermore, due to the diversity of cultures and different levels of development of the countries and their respective healthcare systems, the integration approach must take all the varying specificities of each country into consideration, both cultural and legal.

The final system must perform the different actions and analyses in real-time, adjusting the participating countries’ various time zones. Furthermore, the marketing of the final product must be completed in the participating countries and languages to adapt the different methods to the people’s perception. Finally, the system’s integration must be performed with technical people working on the current systems.

### 4.6. Limitations of Study

This study presents the conceptual foundation of the proposed approach for integrating the 5P-Medicine paradigm within a healthcare system that will be, first and foremost, patient-centric and will implement all of the requirements of the 5P paradigm. This study analyzes the initial concepts and compares them with the current state of affairs while also presenting the current and future challenges this approach will face. The primary study’s shortcoming is that the system is not yet implemented, and all of the analysis is based on the proposed concepts. The direct implications to the patients of such systems can be evident only after the system is implemented and a pilot run is executed in multiple countries.

## 5. Conclusions

Cardiovascular diseases are disorders of the heart and blood vessels and are a significant cause of disability and premature death worldwide. Therefore, individuals at higher risk of developing cardiovascular diseases must be encouraged to maintain active engagement in the healthcare process by promoting the adoption of healthy behaviors and well-being outcomes to prevent premature deaths. Advances in the field of computational intelligence, together with data from connected wearable and smart mobile devices, have made it possible to create recognition systems capable of identifying hidden patterns and valuable information.

In this paper, we presented a new efficiency system for a 5P-Medicine approach to the healthcare systems to manage cardiovascular diseases. This system will facilitate the administration of continual care and offer opportunities for maintaining patients’ active engagement in the care process by promoting patients’ psychological skills and well-being outcomes. The proposed system is defined as a vehicle to enrich patients and stakeholders through the intersection of medical informatics and public health business. As such, through our system, we will promote a new “state of mind” for medical professionals, marked by a global attitude and intention to improve worldwide health.

## Data Availability

Not applicable.
